# Rescue fecal microbiota transplantation for antibiotic-associated diarrhea in critically ill patients

**DOI:** 10.1186/s13054-019-2604-5

**Published:** 2019-10-21

**Authors:** Min Dai, Yafei Liu, Wei Chen, Heena Buch, Yi Shan, Liuhui Chang, Yong Bai, Chen Shen, Xiaoyin Zhang, Yufeng Huo, Dian Huang, Zhou Yang, Zhihang Hu, Xuwei He, Junyu Pan, Lili Hu, Xinfang Pan, Xiangtao Wu, Bin Deng, Zhifeng Li, Bota Cui, Faming Zhang

**Affiliations:** 1 Medical Center for Digestive Diseases, the Second Affiliated Hospital of Nanjing Medical University, 121 Jiang Jia Yuan, Nanjing, 210011 China; 2 Department of Critical Care Medicine, NO.971 Hospital of Chinese People’s Liberation Army Navy, Qingdao, 266000 China; 3 Department of Critical Care Medicine, the Affiliated Yixing Hospital of Jiangsu University, Yixing, 214200 China; 4 Department of Anesthesiology, the Second Affiliated Hospital of Suzhou University, Suzhou, 215000 China; 5 Department of Intensive Care Unit, Renmin Hospital, Hubei University of Medicine, Shiyan, 442000 China; 6 Department of Cardiovascular Surgery, Shenzhen Hospital, Southern Medical University, Shenzhen, 518110 China; 7 Department of Holistic Integrative Medicine, Shenzhen Hospital of Southern Medical University, Shenzhen, 518110 China; 8 Department of Pediatric Intensive Care Unit, the First Affiliated Hospital of Zhengzhou University, Zhengzhou, 450052 China; 9 Department of Critical Care Medicine, Liuzhou General Hospital, Liuzhou, 545006 China; 10 Department of Emergency, the First Affiliated Hospital of Guangxi Medical University, Nanning, 530021 China; 11 Department of Critical Care Medicine, Hefei Hospital Affiliated to Anhui Medical University, Hefei, 230000 China; 12 Department of Critical Care Medicine, Lishui People’s Hospital, the Sixth Affiliated Hospital of Wenzhou Medical University, Lishui, 323000 China; 13 Department of Intensive Care Unit, Qiandongnan People’s Hospital, Kaili, 556000 China; 14 Department of Critical Care Medicine, Shenzhen Hospital, Southern Medical University, Shenzhen, 518110 China; 15 Department of Pediatric Intensive Care Unit, the First Affiliated Hospital of Xinxiang Medical University, Xinxiang, 453100 China; 16 Department of Critical Care Medicine, Zhongnan Hospital of Wuhan University, Wuhan, 430071 China; 17 Key Lab of Holistic Integrative Enterology, Nanjing Medical University, Nanjing, 211100 China

**Keywords:** Fecal microbiota transplantation, Antibiotic-associated diarrhea, Intensive care unit, Critical care, Rescue therapy, Infections, *Clostridium difficile*, Multidrug resistance

## Abstract

**Background:**

Antibiotic-associated diarrhea (AAD) is a risk factor for exacerbating the outcome of critically ill patients. Dysbiosis induced by the exposure to antibiotics reveals the potential therapeutic role of fecal microbiota transplantation (FMT) in these patients. Herein, we aimed to evaluate the safety and potential benefit of rescue FMT for AAD in critically ill patients.

**Methods:**

A series of critically ill patients with AAD received rescue FMT from Chinese fmtBank, from September 2015 to February 2019. Adverse events (AEs) and rescue FMT success which focused on the improvement of abdominal symptoms and post-ICU survival rate during a minimum of 12 weeks follow-up were assessed.

**Results:**

Twenty critically ill patients with AAD underwent rescue FMT, and 18 of them were included for analysis. The mean of Acute Physiology and Chronic Health Evaluation (APACHE) II scores at intensive care unit (ICU) admission was 21.7 ± 8.3 (range 11–37). Thirteen patients received FMT through nasojejunal tube, four through gastroscopy, and one through enema. Patients were treated with four (4.2 ± 2.1, range 2–9) types of antibiotics before and during the onset of AAD. 38.9% (7/18) of patients had FMT-related AEs during follow-up, including increased diarrhea frequency, abdominal pain, increased serum amylase, and fever. Eight deaths unrelated to FMT occurred during follow-up. One hundred percent (2/2) of abdominal pain, 86.7% (13/15) of diarrhea, 69.2% (9/13) of abdominal distention, and 50% (1/2) of hematochezia were improved after FMT. 44.4% (8/18) of patients recovered from abdominal symptoms without recurrence and survived for a minimum of 12 weeks after being discharged from ICU.

**Conclusion:**

In this case series studying the use of FMT in critically ill patients with AAD, good clinical outcomes without infectious complications were observed. These findings could potentially encourage researchers to set up new clinical trials that will provide more insight into the potential benefit and safety of the procedure in the ICU.

**Trial registration:**

ClinicalTrials.gov, Number NCT03895593. Registered 29 March 2019 (retrospectively registered).

## Background

Antibiotic-associated diarrhea (AAD) is defined as otherwise unexplained diarrhea that occurs in association with disrupted gut microbiota caused by administration of antibiotics [1]. AAD occurs in about 5–35% of patients treated with antibiotics [1, 2] and more frequently in critically ill patients [3]. The large volume of watery stools and loss of electrolyte caused by AAD may aggravate the condition of a critically ill patient, leading to higher morbidity, longer hospitalization time, higher medical costs, and worse outcomes [3]. Besides, failure of conventional treatment for AAD is particularly frequent in critically ill patients due to their comorbidities, which has become a huge challenge for critically ill patients and their physicians. A systematic review and meta-analysis showed that probiotics were associated with a reduction of AAD, which indicated the potential preventive role of probiotics for AAD [4]. However, whether the effect can be found in critically ill patients remains uncertain.

*Clostridium difficile* infection (CDI) accounts for about one third of AAD cases and for the vast majority of pseudomembranous colitis (PMC) cases [1]. Fecal microbiota transplantation (FMT), which aims to restore the gut microbiota, has emerged as the most effective alternative for the management of recurrent CDI [5–9]. However, very few reports focus on the application of FMT to the treatment of AAD caused by other or unknown pathogens which accounts for about two thirds of AAD cases. Interestingly, recent reports showed that FMT appeared to be an option for multidrug-resistant organism (MDRO) decolonization [10–12]. The safety of FMT had been ensured both in CDI and MDRO-colonized patients regardless of combination with immunosuppression or immunodeficiency [13, 14]. Our recent studies also reported the safety of FMT in patients with inflammatory bowel disease (IBD) and some of them were immunocompromised [15, 16]. However, patients in the intensive care unit (ICU) were not included in these studies [15, 16]. Whether FMT can be a safe and effective treatment for AAD in critically ill patients requires further investigation. Given that the safety and value of FMT have been identified in severe and recurrent CDI and other gut microbiota dysbiosis-related diseases [9, 14, 17, 18], we aimed to investigate the safety and potential benefit of rescue FMT in critically ill patients with AAD.

## Methods

This case series was performed in 16 ICUs of tertiary hospitals in China including 14 general ICUs and two pediatric ICUs. The rescue FMT therapy course was provided by Chinese fmtBank. This is a part of the registered study of long-term safety and efficacy of rescue FMT for refractory intestinal infections (ClinicalTrials.gov, Number NCT03895593).

### Rescue FMT from Chinese fmtBank

The non-profit organization named Chinese fmtBank (fmtbank.org) provides rescue FMT service for patients with refractory intestinal infection across the whole nation. The protocol and workflow of fmtBank are shown in Additional file 2: Figure S1 and Additional file 3: Table S2. Due to the lack of CDI detection kits in most hospitals in China, the diagnosis of CDI could not be confirmed for all patients, and the reported incidence of refractory CDI in China is lower than that in reality [19–22]. Therefore, the indications of FMT from fmtBank in practice include recurrent or refractory CDI, refractory intestinal infections, and AAD. China Microbiota Transplantation System (CMTS) was set up simultaneously to evaluate long-term safety and efficacy of FMT.

### Donor screening and management

All patients underwent FMT from unrelated universal donors who were 18–24 years old. Other than the initial eligibility screening, regular safety monitoring including dietary guidance, living environment follow-up, and laboratory examinations were scheduled for these donors. Protocol of donor screening and management has been described in our previous studies [16, 23] and is briefly shown in Additional file 3: Table S1.

### Procedure of rescue FMT

Rescue FMT was performed by the cooperated team which consisted of at least two ICU physicians in charge from destination hospitals, two professional FMT clinicians (FZ and BC), two laboratory managers, and one clinical research coordinator from the Second Affiliated Hospital of Nanjing Medical University. The procedure of rescue FMT is shown in Additional file 3: Table S2. Risk factors of serious adverse events (AEs) were evaluated by the rescue team as the exclusion criteria. Excluded patients included those with complete intestinal obstruction, suspected postoperative intestinal leak, and active intestinal fistula within 1 month; without optional FMT delivery way; unable to maintain a suitable position to avoid adverse events such gastric reflux and aspiration; without informed consent from the patient, patient’s family, or legal guardians; and those with other unsuitable conditions for rescue FMT. The rescue team members communicated closely throughout the whole process to ensure the safety of rescue FMT.

### Patients and data collection

**Fig. 1:**
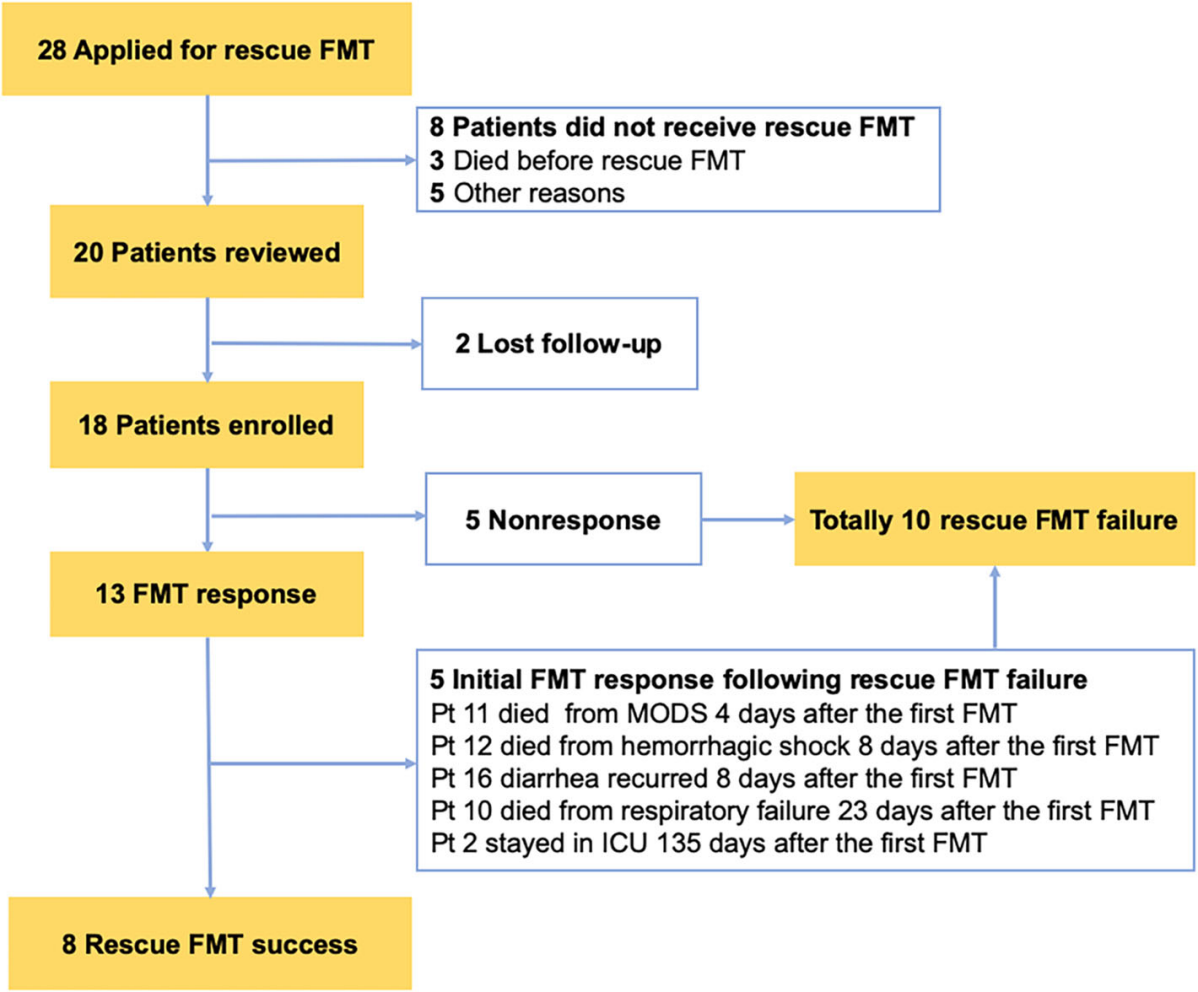
Flow chart and reasons for rescue FMT failure

A database of 28 consecutive critically ill patients with AAD who applied for rescue FMT through their physicians from fmtBank, from September 2015 to February 2019, was reviewed (Fig. 1). Pre-FMT data of patients were collected through the medical records including the demographic characteristics, primary pre-FMT diagnosis from ICU, Acute Physiology and Chronic Health Evaluation (APACHE) II scores, Sequential Organ Failure Assessment (SOFA) score, antibiotic use, probiotic use, abdominal symptoms, and laboratory findings. Part of post-FMT data was collected through the medical records when patients were in the study hospital. Patients were contacted via telephone and asked to finish a questionnaire (Additional file 1) that solicited post-FMT data when they were out of the hospital. A minimum of 12 weeks follow-up after rescue FMT was conducted.

### The outcomes and definitions

AEs and rescue FMT success during a minimum of 12 weeks follow-up were assessed. AEs referred to any new onset of symptoms, the exacerbation of previous symptoms, and abnormal laboratory findings with the use of FMT [24]. The occurrence of AEs in the ICU was recorded on a daily basis, while AEs that occurred out of the ICU were recorded via telephone follow-up or hospital visit. The Common Terminology Criteria for Adverse Events (Version 5.0) was applied to describe the intensity and relativity of AEs with FMT. The intensity of AEs was graded as mild (grade 1), moderate (grade 2), severe (grade 3), life threatening (grade 4), and death (grade 5). Relationship between AEs and FMT was based on clinical judgment and all available information. Consideration of temporal association between FMT exposure and onset of the AEs, and whether the manifestations of the AEs were consistent with known actions or theoretical toxicity of FMT were included. Relationship between AEs and FMT was categorized as definitely related, probably related, possibly related, and unrelated according to a description from Kelly et al. about how to guide investigational new drug application for FMT [24]. AEs were reviewed and classified through voting by the rescue team members.

Rescue FMT success was defined as complete resolution of abdominal symptoms without recurrence and post-ICU survival for a minimum of 12 weeks. FMT response was featured to describe the improvement of abdominal symptoms within 1 week after rescue FMT. FMT nonresponse was defined as persistent abdominal symptoms after rescue FMT. Rescue FMT failure was defined as persistent or recurrent abdominal symptoms or continued ICU stay or death within 12 weeks after rescue FMT. FMT response and nonresponse were evaluated by at least two experienced ICU physicians in charge.

### Statistical analysis

Descriptive analysis was performed in this case series. Patient characteristics were evaluated using mean ± standard deviations (SD) for normally distributed continuous variables, median and range for skewed continuous variables, and proportions for categorical variables. Data were analyzed by IBM SPSS 24.0 or GraphPad 7.0. Analyses included paired *t* test for normally distributed continuous paired data and Wilcoxon matched-pairs signed rank test for skewed continuous paired data. Two tailed *p* value was calculated with each test. *P* values < 0.05 were considered significant.

## Results

### Characteristics of critically ill patients

A total of 20 critically ill patients underwent rescue FMT from fmtBank (Fig. 1). Patients who did not complete the follow-up were excluded from the analysis (*n* = 2). Eighteen patients (Table 1) (age median 55, range 2–91, two patients < 14, male/female 12/6) were included for analysis. The median time of the onset of primary abdominal symptoms before the rescue FMT was 31.5 days (range 8–120 days). The average APACHE II score at ICU admission was 21.7 ± 8.3 (range 11–37). Three patients were tested with *Clostridium difficile* toxin or culture, and one patient had a positive culture of *Clostridium difficile* and the other two were tested toxin negative.

**Table 1:** Patients characteristics, clinical outcomes, and adverse events following FMT

Pt	Age (year)	Sex	Primary ICU diagnosis at the time of rescue FMT	APACHE II score			Extra-intestinal infection sites	Microbiological culture (sample)	Rescue FMT (delivery way, frequency)	FMT response	Adverse events (AEs)			Antibiotic resuming time after FMT	12 weeks survival	Rescue success
				Pre-FMT	3 days after FMT	7 days after FMT					AEs (time after the first FMT)	Grade^a^	Causality between AEs and FMT			
1	25	M	Cerebellar hemorrhage status post craniotomy, catheter associated bloodstream infection	17	12	Discharge	RT, blood	*Klebsiella pneumoniae* (blood)	Gastroscopy, one FMT	Diarrhea and abdominal distention improved	None	–	–	No use	Yes	Yes
2	68	M	Respiratory failure, pneumonia, post-CPR, cerebral infarction, postoperative prostate cancer, PD, GI bleeding	28	26	24	RT	*Acinetobacter baumannii* (sputum)	Nasojejunal tube, two FMTs	Abdominal distention and diarrhea improved	Hematuria (42 days)	–	Unrelated	3 days	Yes	No
											Sudden cardiac arrest (69 days)	–	Unrelated			
											Death (135 days)	–	Unrelated			
3	82	F	Pulmonary infection, encephalatrophy	11	11	11	RT	*Acinetobacter baumannii* (sputum); *Pseudomonas aeruginosa* (sputum); negative (stool)	Nasojejunal tube, one FMT	Nonresponse	Death (52 days)	–	Unrelated	13 days	No	No
4	73	M	Multiple trauma, pulmonary infection	13	Discharge	–	RT	Negative (blood)	Gastroscopy, one FMT	Diarrhea improved	Increased diarrhea frequency (< 1 day)	2	Probably related	7 days	Yes	Yes
5	17	F	Septic shock, MODS, PMC, hypoxic-ischemic encephalopathy, post-CPR	24	19	14	Blood, RT, skin, UT	*Acinetobacter baumannii* (sputum), *E. coli* (blood); *Candida albicans* (stool), *Pseudomonas aeruginosa* (urine)	Nasojejunal tube, four FMTs	Nonresponse after the first FMT, diarrhea and abdominal distention improved after the third FMT	Increased diarrhea frequency (< 1 day)	2	Probably related	8 h	Yes	Yes
6	54	F	Rheumatic heart disease, post-valve replacement	32	34	34	RT, blood, UT	*Candida Albicans* (sputum, urine, stool), *Klebsiella pneumoniae* (sputum), *Enterobacter cloacae* (sputum, blood), *E. coli* (urine), *Enterococcus aureus* (urine)	Nasojejunal tube, two FMTs	Hematochezia alleviated, diarrhea and abdominal pain improved	Abdominal pain (< 1 day)	1	Probably related	29 days	Yes	Yes
7	3	M	Sepsis, septic encephalopathy, MODS, PMC, post-ileostomy	25	31	22	Brain, blood, skin	*Candida albicans* (stool), *Saccharomyces cerevisiae Hansen* (stool)	Nasojejunal tube, one FMT	Diarrhea cured, abdominal distention improved	Increased diarrhea frequency (< 1 day)	1	Probably related	7 days	Yes	Yes
8	27	F	Infective endocarditis, pulmonary infection, septic shock, thoracic empyema, PMC, MODS	39	38	39	Heart, RT, thoracic cavity	*Candida glabrata* (stool)	Nasojejunal tube, two FMTs	Nonresponse	Increased diarrhea frequency (3 days) ^b^	3	Probably related	Continued antibiotic use	No	No
9	27	F	Sepsis, PMC, SLE (severe, active phase, systemic lupus erythematosus), lupus nephritis, pneumonia	12	12	12	Blood, RT	*Enterococcus faecium* (blood), *Pseudomonas aeruginosa* (sputum)	Nasojejunal tube, two FMTs	Transient diarrhea exacerbation, then abdominal distention and diarrhea improved	Hospitalization due to herpes zoster (116 days)	–	Unrelated	24 h	Yes	Yes
10	91	M	Peri-anal abscess, CHD, COPD, cerebral infarction, arrhythmia, atrial fibrillation, NYHA III, cholecystitis, gallstones	20	18	18	Anus, RT	*Pseudomonas aeruginosa* (sputum)	Nasojejunal tube, three FMTs	Diarrhea and abdominal distention improved	Death (23 days)	–	Unrelated	14 days	No	No
11	83	M	COPDAE, respiratory failure, pulmonary encephalopathy, esophagus cancer, hypertension, DM	35	23	Died	RT	*Acinetobacter baumannii* (sputum)	Nasojejunal tube, one FMT	Diarrhea and abdominal distention improved	Death (4 days)	–	Unrelated	20 h	No	No
12	56	M	Septic shock, brain stem infarction, MODS, upper GI bleeding, ischemic necrotizing enteritis? PMC?	25	22	Discharge	Blood, RT	*Pseudomonas aeruginosa* (blood, sputum)	Gastroscopy, one FMT	Diarrhea improved	Upper GI bleeding relapse (6 days)	–	Unrelated	2 days	No	No
											Death (8 days)	–	Unrelated			
13	35	F	Multiple venous thrombosis, abdominal cavity infection, GI bleeding, abdominal hypertension syndrome, PMC, pulmonary infection	22	Died	–	Abdominal cavity, blood, RT	*Acinetobacter baumannii* (blood, sputum)	Enema, four FMTs	Nonresponse	Death (3 days)	–	Unrelated	No use	No	No
14	41	M	Sepsis, septic shock, MODS, post-SAP, pancreatic pseudocyst with acute infection, pulmonary infection, UTI	12	11	7	Pancreas, blood, RT, UT	*Acinetobacter baumannii* (abdominal cavity effusion), *Pseudomonas aeruginosa* (abdominal cavity effusion), *Serratia marcescens* (abdominal cavity effusion)	Nasojejunal tube, two FMTs	Nonresponse	Abdominal pain (< 1 day)	1	Possibly related	24 h	No	No
											Increased Serum amylase (< 1 day)	1	Possibly related			
											Death (46 days)	–	Unrelated			
15	59	M	Multiple trauma, septic shock, PMC, hypertension, CHD	27	17	15	RT, blood, UT	*Acinetobacter baumannii* (sputum, blood, urine, stool)	Nasojejunal tube, two FMTs	Diarrhea and abdominal distention improved	None	–	–	8 h	Yes	Yes
16	2	M	Cardiac arrest, respiratory failure, bronchitis, CNS infection, severe sepsis, severe malnutrition	7	6	7	RT, CNS	Negative (blood, sputum, urine, stool)	Gastroscopy, two FMTs	Diarrhea improved but relapsed 8 days after rescue FMT	Fever (< 1 day)	1	Possibly related	24 h	Yes	No
17	69	F	Post-radical resection of hilar cholangiocarcinoma, post-left hepatectomy	7	7	7	Stoma	*Enterococcus aureus* (stoma secretion)	Nasojejunal tube, one FMT	Nonresponse	None	–	–	6 days	Yes	Yes
18	56	M	Septic shock, refractory CDI, multiple cerebral hemorrhage	14	12	Discharge	RT	*Clostridium difficile* (stool)	Nasojejunal tube, one FMT	Diarrhea, abdominal distention and abdominal pain improved	None	–	–	No use	Yes	Yes

^a^The grade of AEs was evaluated according to the Common Terminology Criteria for Adverse Events, Version 5.0; ^b^< 1 day post the second FMT. *RT* respiratory tract, *PD* Parkinson’s disease, *CPR* cardiopulmonary resuscitation, *GI* gastrointestinal, *MODS* multiple organ dysfunction syndrome, *PMC* pseudomembranous enteritis, *SLE* systemic lupus erythematosus, *CHD* coronary heart disease, *COPD* chronic obstructive pulmonary disease, *COPDAE* COPD acute exacerbation, *DM* diabetes mellitus, *SAP* severe acute pancreatitis, UTI urinary tract infection, *CNS* central nervous system

All patients had complicated extra-intestinal infections (Table 1), 66.7% (12/18) of them had more than one extra-intestinal infection site. The most common infection site was respiratory tract (RT), with 16 (88.9%) patients involved. Sixteen (88.9%) patients had a positive culture of MDRO. For the types of organisms, seven (43.8%) patients were infected with *Acinetobacter baumannii*, six (37.5%) with *Pseudomonas aeruginosa*, and four (25.0%) with *Enterococcus aureus*. Eight (44.4%) patients had sepsis at the time of FMT, and five (27.8%) had multiple organ dysfunction syndrome (MODS).

### Antibiotic and probiotic use before and after FMT

**Fig. 2:**
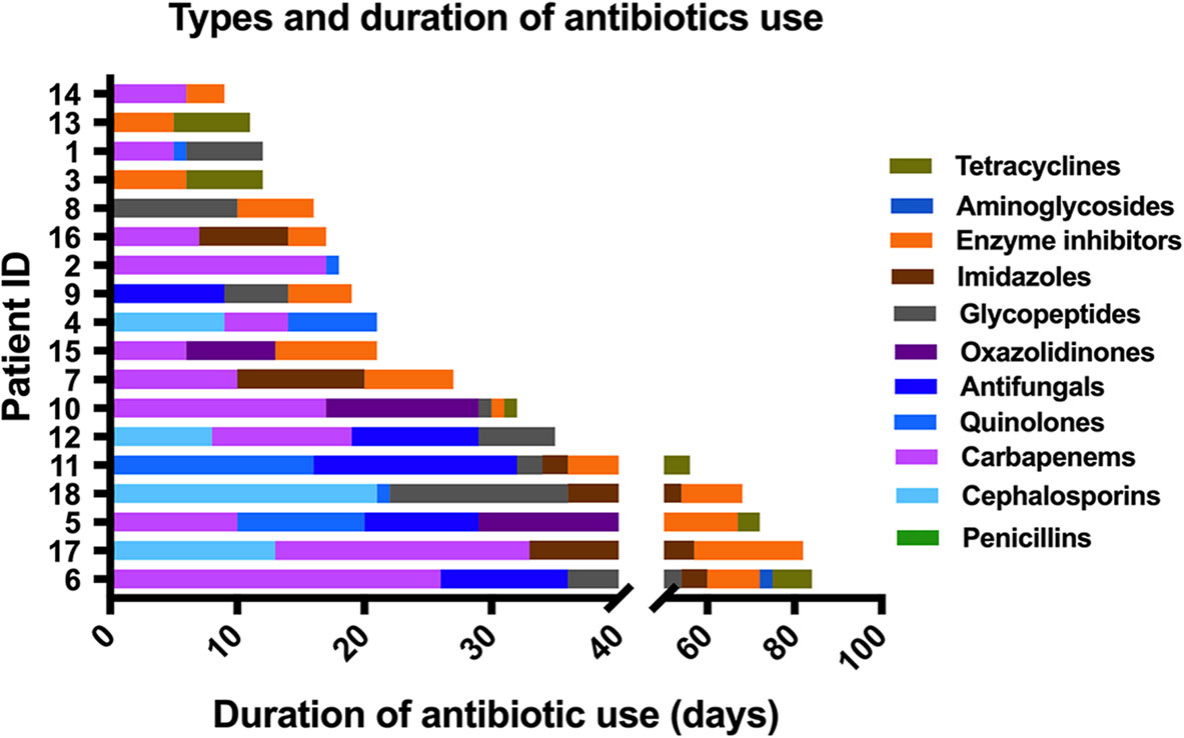
Types and duration of antibiotic use before rescue FMT (*n* = 18)

Patients were treated with a mean of four (4.2 ± 2.1, range 2–9) types of antibiotic before and during the onset of AAD. The types and duration of antibiotic use are shown in Fig. 2. Six (33.3%) patients were medicated with vancomycin for the treatment of AAD before FMT, two (11.1%) with metronidazole, and two (11.1%) with vancomycin and metronidazole. Seventeen (94.4%) patients discontinued the antibiotic use 12–24 h before FMT. Besides, the majority of patients (83.3%) continued antibiotic treatment after FMT. Seven (38.9%) patients reused antibiotics within 24 h after FMT, 12 (66.7%) within one week after FMT. The antibiotic resuming time after FMT is listed in Table 1. Sixteen (88.9%) patients were given probiotics for the prevention or treatment for AAD before and during the AAD onset, and 13 (72.2%) patients took more than one type of probiotics.

### Adverse events following rescue FMT

In total, 33 FMTs were performed on 18 critically ill patients (the frequency and delivery way of FMT are listed in Table 1). Among them, seven (7/18, 38.9%) patients had FMT-related AEs during follow-up. And Pt 14 had two FMT-related AEs. The most common FMT-related AEs were increased diarrhea frequency and abdominal pain. All AEs are listed in Table 1. Eight deaths occurred during the follow-up, which were categorized unrelated to FMT (Table 1). The core causes of death are listed in Additional file 3: Table S3. One patient (Pt 10) developed diarrhea exacerbation with increase of diarrhea frequency within 24 h after the first FMT due to the low temperature of fecal microbiota suspension. But the second FMT improved the diarrhea which was reflected on the improvement of stool consistency and decrease of diarrhea frequency. This patient recovered from severe sepsis and PMC and was finally discharged from ICU. She came back to the hospital for a checkup for pneumonia 8 weeks and 16 weeks after FMT. Chest computed tomography indicated the resolution of pneumonia. But she developed herpes zoster and was hospitalized 116 days after the first FMT, which was considered to be unrelated to FMT.

### Clinical outcomes following rescue FMT

**Fig. 3:**
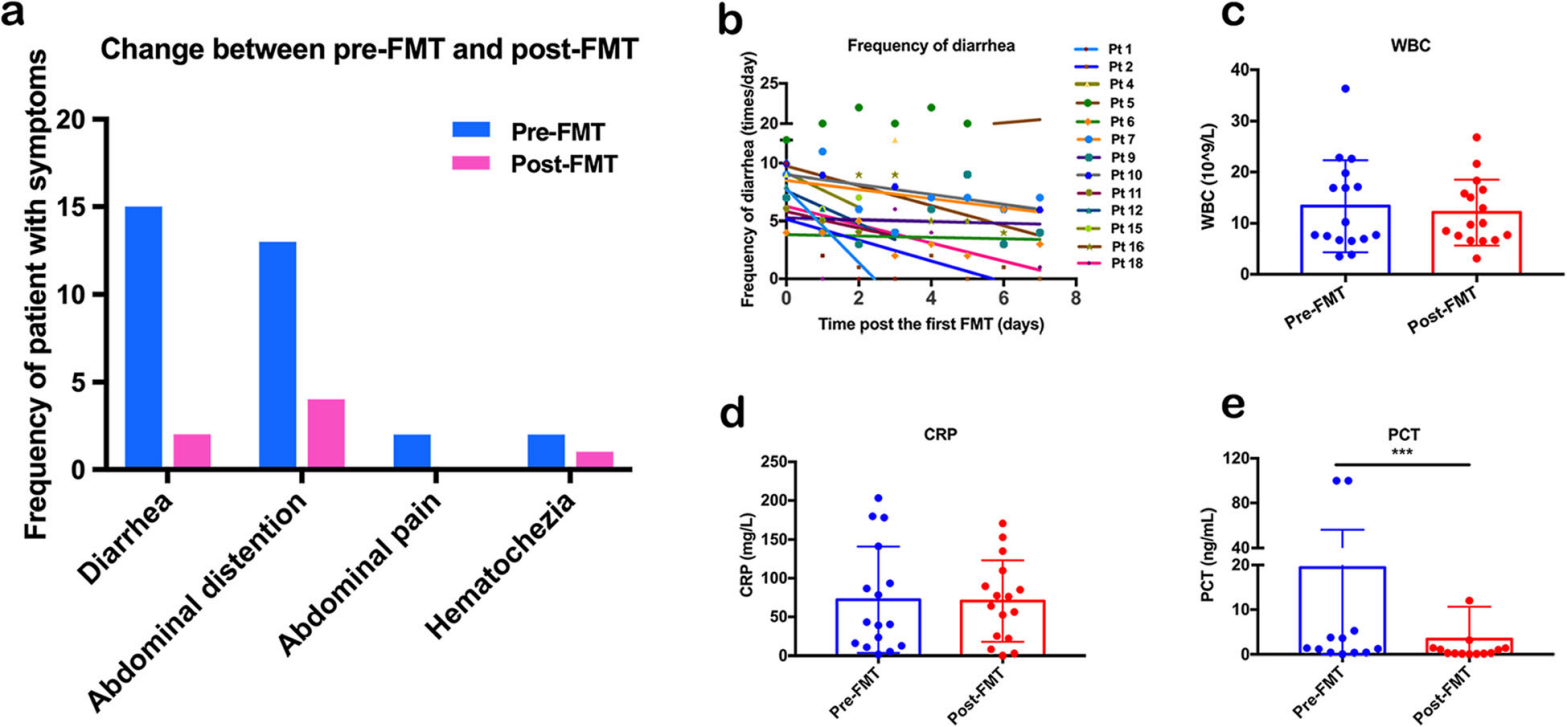
Abdominal symptoms and laboratory markers of inflammation. **a** Frequency of patients with abdominal symptoms pre-FMT and post-FMT (*n* = 18). **b** Frequency of diarrhea of patients who were responsive to rescue FMT within 1 week post the first FMT (*n* = 13). **c–e** Level of WBC count, CRP, and PCT (*p* = 0.0005) pre-FMT and 1 week post the first FMT (*n* = 18)

Clinical outcomes following rescue FMT for AAD in critically ill patients are shown in Table 1. 72.2% (13/18) of patients achieved FMT response within one week after FMT (Fig. 3a). Among 15 patients with primary abdominal symptoms of diarrhea, 86.7% (13/15) of them achieved improvement (Fig. 3b). 69.2% (9/13) of patients achieved improvement of abdominal distention including two patients as primary abdominal symptoms and seven as secondary abdominal symptoms. And 100% (2/2) of patients’ abdominal pain was improved, and 50% (1/2) of hematochezia was alleviated. In addition, the laboratory findings showed that WBC count, CRP, and PCT after FMT were decreased to a certain degree, though a significant difference can only be seen in PCT (*p* = 0.0005), compared with pre-FMT conditions (Fig. 3c–e).

Rescue FMT success was observed in eight (44.4%) patients, and the median length of follow-up was 50.6 weeks (range 12–191.8 weeks, by May 2019). Reasons for rescue FMT failure are shown in Fig. 1. Among the eight rescue success patients, five patients had bacteremia, including *Klebsiella pneumoniae*, *Escherichia coli*, *Enterobacter cloacae*, *Enterococcus faecium*, and *Acinetobacter baumannii*. Pt 1 was discharged from ICU 4 days after FMT and transferred to a recovery room without a re-examination of microbiological culture. Pt 5 was checked for the microbiological culture of blood 1 month after FMT, and the result indicated MDRO decolonization. The MDROs colonizing in Pt 7, 9, and 15 were not cleared before they were discharged from the ICU, and the final post-ICU results were not detected.

## Discussion

This case series focused on the safety and potential benefit of rescue FMT for AAD in critically ill patients. The results showed that 38.9% (7/18) of patients had FMT-related AEs during the minimum of 12 weeks follow-up. No FMT-related death or infective complications occurred. 72.2% (13/18) of patients achieved improvement of abdominal symptoms within one week, and 44.4% (8/18) of patients acquired rescue FMT success.

The potential serious AEs such as infective complications following FMT in critically ill patients are the primary concern of clinicians, which have so far limited the use of FMT [25, 26]. There were a few case reports and case series about the application of FMT in critically ill patients in the ICU [27–31]. Less attention was paid particularly to the incidence of AEs of FMT in critically ill patients [28, 30]. In this series, 38.9% (7/18) of patients had FMT-related AEs, which was higher than that in previous studies of FMT for non-ICU patients [7, 15, 16, 18]. This may be attributed to that the patients in the ICU were more sensitive to the interventional changes. Importantly, the US Food and Drug Administration (FDA) in June 2019 issued a serious FMT-related AE that may be attributed to the improper donor screening. This safety event highlighted the importance of strict criteria for donor screen [32, 33] and assessment on status of recipients including their age, immune fuction, and nutritional status [34]. In addition, the methodology of fecal microbiota preparation might be an important factor affecting safety [33, 34]. Our recent studies based on IBD patients indicated that purification of microbiota from donated stool in a GMP-level lab with an automatic machine contributed the significantly decreased AEs [15, 16, 33, 34].

For the heterogeneity of patients in the ICU, more data were needed to identify which subset of ICU patients would potentially benefit more from FMT with less AEs. Moreover, the procedure of rescue FMT for critically ill patients based on the multidisciplinary cooperation, individualized therapy strategy such as route of administration and antibiotic use, and management for AEs, was the most important part for ensuing safety of FMT. A multidisciplinary team has been set up in fmtBank including gastroenterologists who are professional in FMT, endoscopists, and infectious disease physicians as recommended by Cammarota et al. [26, 32]. The team cooperated with the patients’ physicians to perform FMT and monitor for the short-term (within one month post-FMT) and long-term (over one month post-FMT) AEs [16] to keep the safety of FMT as far as possible. The majority of FMT-related AEs in the present case series were mild to moderate and < 1 day after the first FMT except one severe AE in Pt 8. This patient’s condition progressed very quickly and developed severe diarrhea with blood and lots of necrotic intestinal mucosa mixed in the stool 17 days after the primary diarrhea onset. Her APACHE II score at the first 24 h of ICU admission was 39 (Additional file 4), which indicated the disease severity and high risk of mortality [35]. Multiple conventional therapies were used before rescue FMT but could not prevent the progression from AAD to PMC. Rescue FMT was used as the last attempt to save life in this patient but things did not work out.

Rescue FMT showed promising benefits for AAD in critically ill patients. 100% (2/2) of abdominal pain, 86.7% (13/15) of diarrhea, 69.2% (9/13) of abdominal distention, and 50% (1/2) of hematochezia can be alleviated by FMT. These results were similar to those of a recent case series which included nine critically ill patients with severe CDI [30]. It indicated that following FMT there was marked improvement in clinical status with resolution of diarrhea and reduction in abdominal distention and pain [30]. Alleviation of these abdominal symptoms may improve life quality of the critically ill patients and provided chances for other treatments. Interestingly, among the patients achieving rescue FMT success, five of them had bacteremia caused by enteric bacteria. One patient (Pt 5) had the clearance of bacteremia of *E. coli* after FMT with application of antibiotic treatment. Four patients were free of symptoms of bacteremia though there were no negative cultures of blood. Besides, Li et al. [36] described a 29-year-old woman with bacteremia of *Acinetobacter baumannii* whose microbiological culture of blood was negative 1 day after FMT without using antibiotics. Singh et al. [37] reported that three out of 15 (20%) patients carrying extended spectrum beta lactamase (ESBL)-producing *Enterobacteriaceae* were ESBL-negative at 1, 2, and 4 weeks after the first FMT, while six out of 15 (40%) were negative after the second transplant. But a recent randomized controlled study showed limited benefit of antibiotics followed by FMT for MDRO decolonization [12]. More prospective data are needed. And the timing for discontinuing and resuming antibiotics may be another important question. Continued antibiotic use during FMT and early resuming of antibiotics after FMT may be risk factors for multiple FMTs in this series.

Among the patients who achieved rescue FMT success, 62.5% (5/8) of them reused antibiotics within 1 week after the first FMT, but their diarrhea did not exacerbate or recur. Meanwhile, the complicated infections were alleviated. On the one hand, restoration of gut microbiota through FMT can repair the integrity of intestinal mucosal and prevent bacteria translation to acquire the improvement of diarrhea and reduction of inflammation [38]. On the other hand, gut microbiota has significant interactions on immunity [38]. Combination of conventional treatments and FMT may be more effective than either. Cui et al. described the concept of step-up FMT strategy which included step 1: single FMT; step 2: multiple FMTs; and step 3: FMT followed by steroids, which helped 57.1% of steroid-dependent ulcerative colitis patients maintain steroid dependence [17]. Fischer et al. suggested that additional antibiotic treatment for CDI after FMT, followed by a second FMT, may improve outcomes in patients with severe CDI [39], which is similar to the concept of step 3 in the step-up FMT strategy [17, 40].

Several studies showed the prevention and treatment value of probiotics for AAD [4, 41–43]. It is worth mentioning that 88.9% of patients in this case series were given probiotics to prevent or treat AAD. However, one single type or several types of probiotics seemed to have limited effects in critically ill patients with AAD. Compared with probiotics, FMT comes with risks of pathogen transmission from the donor and an increase of AEs but might be a better method for complete restoration of gut microbiota [38]. Nonetheless, further studies should aim at identifying the precise bacterial strains and specific functions, and two or more bacterial strains’ co-effect named selective microbiota transplantation (SMT) might be the new direction [33].

The limitation of this series is the small sample size of subjects and lack of control group. It is difficult to select critically ill patients for potential FMT trials because of the heterogeneity, so the bias cannot be avoided for the narrow subset of patients. In short of microbiota sequencing data, we are unable to provide direct evidence for dynamic changes of gut microbiota in critically ill patients before and after FMT. Further insights into the potential of precise bacterial strains and their specific functions as the predictive factors for FMT success and failure might be given by the integrated microbial analysis. The potential effect of FMT on MDRO decolonization also needs to be answered. Elaborately designed studies with a large sample size are needed.

## Conclusion

In this case series studying the use of FMT in critically ill patients with AAD, good clinical outcomes without infectious complications were observed. These findings could potentially encourage researchers to set up new clinical trials that will provide more insight into the potential benefit and safety of the procedure in the ICU.

## Supplementary information

**Additional file 1.** Rescue Fecal Microbiota Transplantation (FMT) Follow-Up Survey.

**Additional file 2. Figure S1.** Work flow of rescue FMT in Chinese fmtBank.

**Additional file 3. Table S1.** Protocol of donor screening, donor management and fecal microbiota preparation in Chinese fmtBank. **Table S2.** Procedure of rescue FMT for critically ill patients with AAD. **Table S3.** Time and Core causes of death during follow-up.

**Additional file 4.** Sequential Organ Failure Assessment (SOFA) score before and after FMT.

## Data Availability

The data used and analyzed in this case series are available from the corresponding author on reasonable request.

## Abbreviations

AAD: Antibiotic-associated diarrhea
CDI: *Clostridium difficile* infection
PMC: Pseudomembranous colitis
FMT: Fecal microbiota transplantation
MDRO: Multidrug-resistant organisms
IBD: Inflammatory bowel disease
ICU: Intensive care unit
CMTS: China Microbiota Transplantation System
AE: Adverse event
APACHE: Acute Physiology and Chronic Health Evaluation
SD: Standard deviations
RT: Respiratory tract
PD: Parkinson’s disease
MODS: Multiple organ dysfunction syndrome
GI: Gastrointestinal
SLE: Systemic lupus erythematosus
CHD: Coronary heart disease
COPD: Chronic obstructive pulmonary disease
COPDAE: COPD acute exacerbation
DM: Diabetes mellitus
UTI: Urinary tract infection
CNS: Central nervous system
CPR: Cardiopulmonary resuscitation
WBC: White blood cell
CRP: C-reactive protein
PCT: Procalcitonin
FDA: Food and Drug Administration
ESBL: Extended spectrum beta lactamase
SMT: Selective microbiota transplantation
