# Supplementary material for: Rescue fecal microbiota transplantation for antibiotic-associated diarrhea in critically ill patients
Source: Crit Care. 2019 Oct 21;23:324. doi: 10.1186/s13054-019-2604-5 (PMC6805332; doi:10.1186/s13054-019-2604-5)
Supplement: Supplementary file 1 — Additional file 1. Rescue Fecal Microbiota Transplantation (FMT) Follow-Up Survey. [file 13054_2019_2604_MOESM1_ESM.docx]

**Rescue Fecal Microbiota Transplantation (FMT) Follow-Up Survey**

Patient ID:

Interviewee: Patient , Patient family member , Other relatives

Current status: Hospitalized (ICU , Other departments ),

Community care ,

Other status

Date of interview:

Date of the first FMT:

Interviewer:

1. Regarding the following symptoms, check which applies to you:

Diarrhea: Yes (resolved improved did not improve )

No

Abdominal distention: Yes (resolved improved did not improve ) No

Abdominal pain: Yes (resolved improved did not improve )

No

Hematochezia: Yes (resolved improved did not improve )

No

1. Did you have a recurrence of the above symptoms after the rescue FMT?
2. No
3. Yes

If yes, which symptom recurred and how long after the first FMT did it recur?

1. Have you reused antibiotics after FMT rescue therapy?
2. No
3. Yes

If yes, how long after the first FMT did you reuse antibiotics?

1. Have you re-tested the bacteria culture which you had during the ICU stay?
2. No
3. Yes

If yes, did the culture of bacteria turn negative?

1. Have you had any new medical conditions including (new onset of symptoms, the exacerbation of previous symptoms or abnormal laboratory findings; unplanned hospitalization, life-threating events or other medical events) since the first FMT?
2. No
3. Yes

If yes, please specify:
