# Supplementary figures and images for: Rescue fecal microbiota transplantation for antibiotic-associated diarrhea in critically ill patients
Source: Crit Care. 2019 Oct 21;23:324. doi: 10.1186/s13054-019-2604-5 (PMC6805332; doi:10.1186/s13054-019-2604-5)

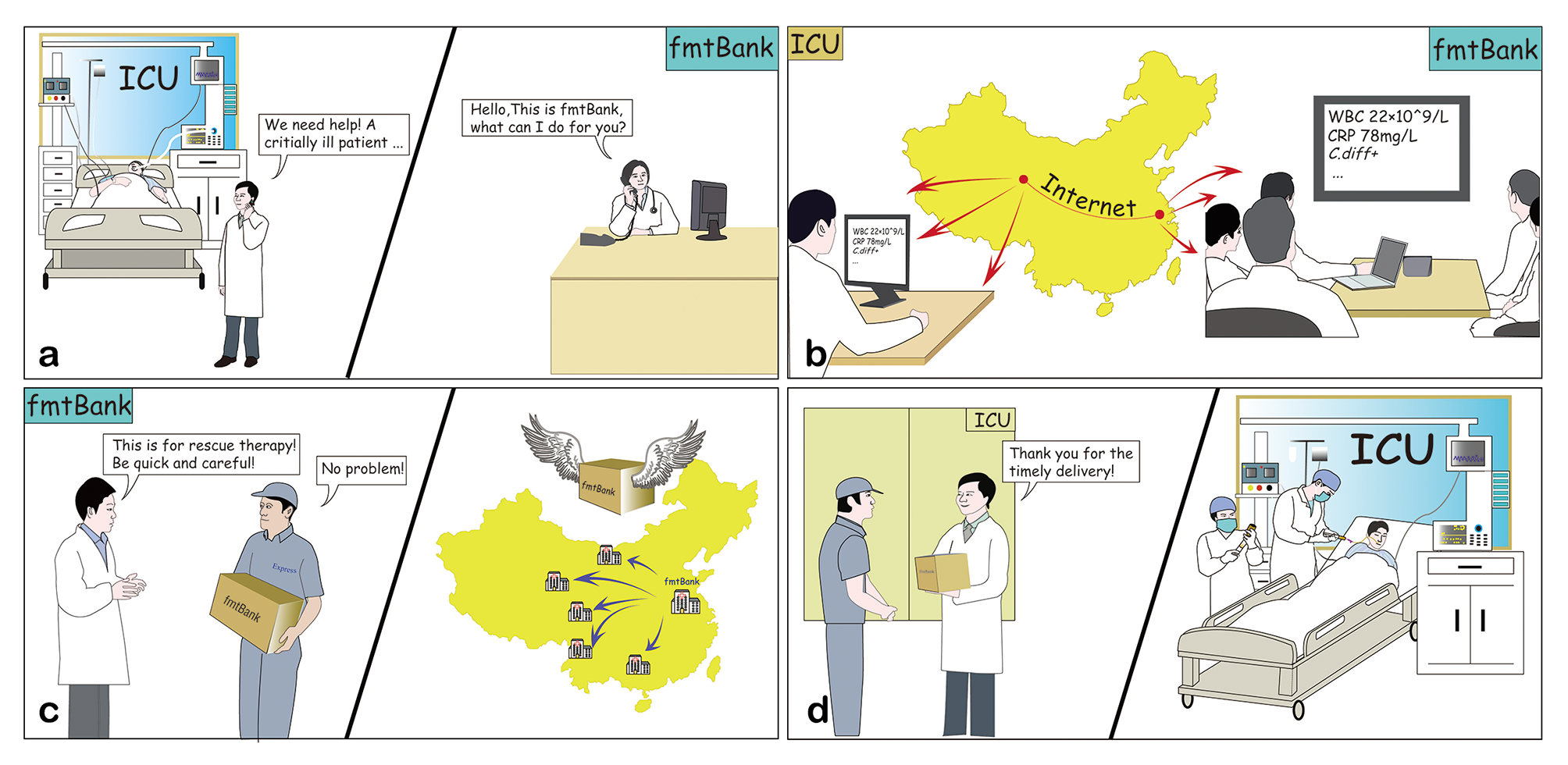

Supplement: Supplementary file 2 — Additional file 2. Figure S1. Work flow of rescue FMT in Chinese fmtBank. [file 13054_2019_2604_MOESM2_ESM.tif]
