# Supplementary material for: Rescue fecal microbiota transplantation for antibiotic-associated diarrhea in critically ill patients
Source: Crit Care. 2019 Oct 21;23:324. doi: 10.1186/s13054-019-2604-5 (PMC6805332; doi:10.1186/s13054-019-2604-5)
Supplement: Supplementary file 3 — Additional file 3. Table S1. Protocol of donor screening, donor management and fecal microbiota preparation in Chinese fmtBank. Table S2. Procedure of rescue FMT for critically ill patients with AAD. Table S3. Time and Core causes of death during follow-up. [file 13054_2019_2604_MOESM3_ESM.docx]

**Table S1. Protocol of donor screening, donor management and fecal microbiota preparation in Chinese fmtBank**

| **Section 1. Protocol of donor screening in Chinese fmtBank** (Donor screening in Chinese fmtBank is based on eight dimensions: age, physiology, pathology, psychology, veracity, time, living environment and recipients). [1, 2] | |
| --- | --- |
| **Step 1** | **Giving lecture about FMT and Chinese fmtBank to young students at Nanjing Medical University** |
| **Step 2** | **Questionnaire screening** |
|  | Inclusion criteria: |
|  | 1. Willing to finish questionnaire screening and sign informed consent; |
|  | 1. Age range 18 to 24 years old; |
|  | 1. Normal body mass index (18-24) and regular bowel habits; |
|  | Exclusion criteria: |
|  | 1. History of drug |
|  | Received antibiotics, probiotics of pharmaceutical grade, laxative within 3 months. |
|  | 1. History of diseases |
|  | History of all known infection diseases, gastrointestinal diseases (IBD, IBS, chronic diarrhea, constipation and others), allergy, gastrointestinal surgery, autoimmune diseases, malignant tumor, mental illness, diabetes, hypertension, hyperlipidemia, and other diseases or conditions associated with changes in gut microbiota. |
|  | 1. Family history |
|  | First degree relative with gastrointestinal diseases (IBD, IBS, chronic diarrhea, constipation, carcinoma), diabetes, metabolic syndrome, mental diseases, genetic diseases; |
|  | 1. Sex behaviors |
|  | With sexual activity during past 6 months. |
| **Step 3** | **Face to face interview** |
|  | Inclusion criteria: |
|  | 1. Willing to donate feces and cooperate with management; |
|  | 1. Normal sleep quality, diet habits, physical exercise. |
|  | Exclusion criteria: |
|  | 1. Persons judged to have questionable integrity; |
|  | 1. Any anxiety, depression and other abnormal psychology situation; |
|  | 1. Living in geographic extremes such as regions of high altitude, high temperature, cold, high humidity, severely polluted areas; |
|  | 1. Exposure to epidemic area and still in the window stage; |
|  | 1. Smoking or drug/alcohol abuse. |
| **Step 4** | **Laboratory examination** |
|  | Inclusion criteria: |
|  | Normal blood cell count, CRP, ESR, immunoglobulin (IgA, IgM, IgG, IgE), serum lipid, FPG and other biochemical results; |
|  | Exclusion criteria: |
|  | 1. Blood tests: Positive finding of HIV-1 or HIV-2 (anti-HIV), HBV (HBsAg + anti-HBc IgM), HCV (anti-HCV), Syphilis (Ig), TORCH (Cytomegalovirus IgM, Rubella virus IgM, Herpes simplex virus types 1 and 2 IgM, Toxoplasma gondii IgM), HAV (IgM + IgG), HEV (IgM + IgG), EBV (IgM + IgG); |
|  | 1. Stool tests: Positive finding of *Clostridium difficile* toxin A/B, Shigella spp., *Salmonella* spp., *Campylobacter* spp., *Escherichia coli* O157 H7, Shiga-producing *Escherichia coli,* *Yersinia enterocolitis*; MDROs: Vancomycin-resistant *Enterococci* spp., Carbapenem-resistant *Enterobacteriaceae*, ESBL-producing *E. coli*, Methicillin-resistant *Staphylococcus aureus*; *Aeromonas* spp., *Plesiomonas* spp., *Yesinia* spp., *Vibrio* spp.; Virus: Rotavirus, Adenovirus, HEV, Polio virus; Parasites: Cryptosporidium, Entamoeba histolytica, Giardia lamblia, , Microspore, Isosporiasis; Fecal occult blood test. |
| **Section 2. Protocol of donor management in Chinese fmtBank** | |
| **Step 1** | **Diet guideline** |
|  | A nutritious, balanced diet is recommended, seafood, spicy and potential unclean food is unrecommended. |
| **Step 2** | **Habits and living condition guideline** |
|  | Regular sleep, physical exercise is recommended, staying up late and overwork are not recommended; |
|  | Relax and normal mood are recommended, anxiety or depression mood is not recommended. |
| **Step 3** | **Regular monitoring** |
|  | Regular laboratory examination for every 3-6 months, additional examination for leaving the regular residence; |
|  | Transient diarrhea, bloating or other gastrointestinal discomfort lead to pause to donate for at least 2 weeks and re-donate after normal laboratory examination;  Sexual activity or high risk of activity related to disease spreading. |
| **Section 3. Protocol of fecal microbiota preparation in Chinese fmtBank** (The whole fecal microbiota preparation is processed in a GMP-level lab.) | |
| **Step 1** | Feces collection: Collect all fresh feces with a sterilized container which is designed to match the automatic purification machine; |
| **Step 2** | Fecal suspension preparation and filtration: Install the container into the automatic purification system (GenFMTer, FMT Medical, Nanjing, China), add 500-1000 mL 0.9% saline into the container, and the filtration automatically processes within the machine. |
| **Step 3** | Centrifugation: Put the fecal suspension into a 50 mL sterilizing centrifuge tube, and centrifuge at 2000g for 3min;  Washing: Remove the supernatant, add normal saline to 50 mL, mix gently and centrifuge at 2000g for 3 min;  Repeat the “washing” step for 3-5 times;  Discard the most of supernatant, and the ultimate deposit is the purified fecal microbiota. |
| **Step 4** | Fecal microbiota suspension: Dilute the microbiota with 1.5-fold 0.9% normal saline; The fresh concentrated fecal microbiota suspension must be administered to the patient intestine immediately. |
| **Step 5** | Storage: The suspension also can be stored with 10% sterile pharmaceutical grade glycerol at -80°C. The information of donor ID number, dosage, preparation date and preservation condition are labeled. |

IBD: Inflammatory bowel disease; IBS: irritable bowel disease; CRP: C-active protein; ESR: erythrocyte sedimentation rate; FPG: fasting plasma glucose; HIV: human immunodeficiency virus; HBV: hepatitis B virus; HCV: hepatitis C virus; HEV: hepatitis E virus; EBV: Epstein-Barr virus; MDROs: multidrug-resistant organisms; ESBL: extended-spectrum beta-lactamase; GMP: good manufacturing practices.

1. Ding X, Li Q, Li P, Zhang T, Cui B, Ji G, Lu X, Zhang F: **Long-Term Safety and Efficacy of Fecal Microbiota Transplant in Active Ulcerative Colitis**. *Drug Saf* 2019, **42**(7):869-880.

2. Cui B, Feng Q, Wang H, Wang M, Peng Z, Li P, Huang G, Liu Z, Wu P, Fan Z *et al*: **Fecal microbiota transplantation through mid-gut for refractory Crohn's disease: safety, feasibility, and efficacy trial results**. *J Gastroenterol Hepatol* 2015, **30**(1):51-58.

**Table S2. Procedure of rescue FMT for critically ill patients with AAD**

| **Procedure of recue FMT for critically ill patients with AAD** | |
| --- | --- |
| **ICU clinicians applying** | 1. Contact with Chinese fmtBank; |
|  | 1. Submit the application documents including patients’ diagnosis, laboratory test results and other description of disease process. |
| **FmtBank reviewing** | 1. Indication for the rescue FMT; |
|  | 1. Exclusion criteria for critically ill patients; |
|  | 1. Approval after reviewing. |
| **Rescue team establishment** | 1. Further talk on the risk for adverse events and potential benefit; |
|  | 1. Choose optimal delivery way and position; |
|  | 1. Obtain written informed consent from patients or pediatric patients’ parents or legal guardians. |
| **Delivering fecal microbiota** | Chinese fmtBank delivers prepared fecal microbiota: sending date, dose, donor ID number, recipient name and destination hospital are recorded. |
| **Fecal microbiota transport** | 1. Fecal microbiota preservation: the bottled suspension is placed in an insulated box with 5-10 kg dry ice; |
|  | 1. Transport: the package is transport through express to the destination hospital. |
| **Patient preparation** | 1. Support therapy is given according to patients’ condition; |
|  | 1. Antibiotic is recommended to be discontinued 12-24 h before FMT; |
|  | 1. Delivery way: nasojejunal tube is recommended to be insert into the jejunum under X-ray guidance (other delivery way is chosen based on the advice from rescue team). |
| **Protocol of FMT** | 1. Rewarming: the suspension is rewarmed by water bath to 37- 39 ˚C; |
|  | 1. Infusion: 150-200 mL liquid suspension (include ~ 60cm^3^ fecal flora and ~ 100 mL normal saline, children < 14 years receive ~ 1/3 of the total volume) is transplanted into patient’s intestinal through nasojejunal tube in a head high position. |
| **Follow-up** | Laboratory tests including blood routine and CRP, and changes of abdominal symptoms are assessed daily for the first week, weekly after 1 week. |

**Table S3. Time and Core causes of death during follow-up.**

| **Pt** | **Days after the first FMT (d)** | **Primary diagnosis at ICU administration** | **Core cause of death** |
| --- | --- | --- | --- |
| 2 | 135 | Respiratory failure, pneumonia, post-CPR, cerebral infarction, postoperative prostate cancer, PD, GI bleeding | Respiratory failure, heart failure, GI bleeding |
| 3 | 52 | Pulmonary infection, encephalatrophy | Respiratory failure, heart failure, pulmonary infection and brain tumor |
| 8 | 7 | Infective endocarditis, pulmonary infection, septic shock, thoracic empyema, PMC, MODS | Pulmonary infection, sepsis, infective endocarditis, shock (septic and cardiogenic), MODS, type Ⅰ respiratory failure, PMC, abandon treatment |
| 10 | 23 | Crissum abscess, CHD, COPD, cerebral infarction, arrhythmia, atrial fibrillation, NYHA Ⅲ, cholecystitis, gallstones | COPDAE causing respiratory failure and heart failure |
| 11 | 4 | COPDAE, respiratory failure, pulmonary encephalopathy, esophagus cancer, hypertension, DM | MODS, severe pulmonary infection and blood infection |
| 12 | 8 | Septic shock, brain stem infarction, MODS, upper GI-bleeding, ischemic necrotizing enteritis? PMC? | Hemorrhagic shock, MODS, abandon treatment |
| 13 | 3 | Multiple venous thrombosis, abdominal cavity infection, GI-bleeding, abdominal hypertension syndrome, PMC, pulmonary infection | Heart failure, abdominal cavity infection, abdominal hypertension syndrome |
| 14 | 46 | Sepsis, septic shock, MODS, post-SAP, pancreatic pseudocyst with acute infection, pulmonary infection, UTI | MODS, GI bleeding, severe pancreatitis |

CPR: cardiopulmonary resuscitation; PD: Parkinson’s disease; GI: gastrointestinal; PMC: pseudomembranous enteritis; MODS: multiple organ dysfunction syndrome; CHD: coronary heart disease; COPDAE: chronic obstructive pulmonary disease acute exacerbation; DM: diabetes mellitus; UTI: urinary tract infection.
