# Supplementary material for: Rescue fecal microbiota transplantation for antibiotic-associated diarrhea in critically ill patients
Source: Crit Care. 2019 Oct 21;23:324. doi: 10.1186/s13054-019-2604-5 (PMC6805332; doi:10.1186/s13054-019-2604-5)
Supplement: Supplementary file 4 — Additional file 4. Sequential Organ Failure Assessment (SOFA) score before and after FMT. [file 13054_2019_2604_MOESM4_ESM.docx]

**Additional file 4. Sequential Organ Failure Assessment (SOFA) score before and after FMT**

| **Pt** | **SOFA score** | | | | | | |
| --- | --- | --- | --- | --- | --- | --- | --- |
|  | **Within 24 h of ICU admission** | **Pre-FMT** | **3 days post-FMT** | **7 days post-FMT** | **2 weeks post-FMT** | **4 weeks post-FMT** | **Rescue success** |
| 1 | 6 | 6 | 5 | - | - | - | Yes |
| 2 | 6 | 7 | 6 | 7 | 6 | 9 | No |
| 3 | 1 | 1 | 2 | 3 | 2 | 2 | No |
| 4 | 3 | 3 | 3 | 3 | - | - | Yes |
| 5 | 15 | 12 | 11 | 11 | 14 | 6 | Yes |
| 6 | 10 | 12 | 8 | 4 | 1 | 0 | Yes |
| 7 | 3 | 2 | 1 | 1 | 1 | 0 | Yes |
| 8 | 8 | 14 | 15 | - | - | - | No |
| 9 | 8 | 3 | 1 | 0 | - | - | Yes |
| 10 | 0 | 4 | 1 | 1 | 1 | - | No |
| 11 | 9 | 20 | 20 | 24 | - | - | No |
| 12 | 15 | 12 | 11 | 5 | 11 | - | No |
| 13 | 2 | 17 | - | - | - | - | No |
| 14 | 6 | 3 | 5 | 7 | 6 | 9 | No |
| 15 | 7 | 20 | 11 | - | - | - | Yes |
| 16 | 1 | 0 | 0 | 0 | - | - | No |
| 17 | 4 | 3 | 3 | 3 | 1 | - | No |
| 18 | 4 | 4 | 4 | - | - | - | Yes |

-: discharge.
